# Technology‐enabled patient care in medical radiation sciences: the two sides of the coin

**DOI:** 10.1002/jmrs.807

**Published:** 2024-06-24

**Authors:** Christina Malamateniou

**Affiliations:** ^1^ Department of Midwifery & Radiography, School of Health and Psychological Sciences, City University of London London UK; ^2^ Discipline of Medical Imaging and Radiation Therapy, College of Medicine and Health University College Cork Cork Ireland; ^3^ European Federation of Radiographer Societies Cumiera Portugal; ^4^ European Society of Medical Imaging Informatics Vienna Austria

## Abstract

This is an exciting time to be working in healthcare and medical radiation sciences. This article discusses the potential benefits and risks of new technological interventions for patient benefit and outlines the need for co‐production, governance and education to ensure these are used for advancing patients' well‐being.

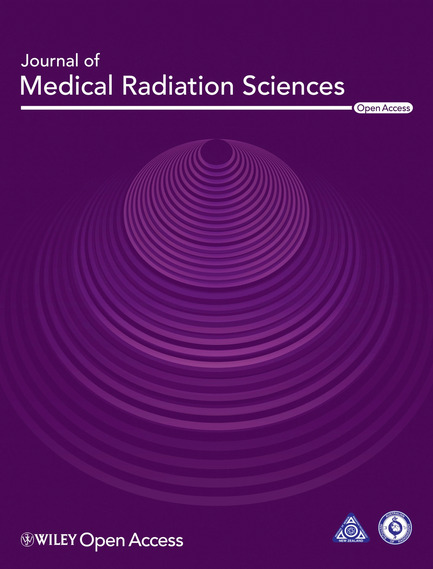

## Introduction: Healthcare Innovation in Medical Radiation Sciences (MRS): Part of Our Professional Identity

Technological innovation has been intertwined with MRS since its inception. The dyadic nature of the role and professional identity of a radiographer, radiation therapist and nuclear medicine technologist (or medical radiation practitioner as more commonly known in Australia and New Zealand) commands both technological acumen and patient care expertise.^1^ More specifically, medical radiation practitioners are expert healthcare professionals who use technology in medical imaging and radiation therapy to benefit the patients through improvement of diagnosis and/or treatment.^2^ They achieve these while ensuring patient safety and an optimal patient experience. This duality of our role (technical expertise coupled with patient care values) is a commonly accepted principle we are educated with, and practise within, one we cannot operate without and that differentiates us from other healthcare professions.

## MRS Innovation as Key to Person‐Centred Care

There is an abundance of technological innovation within the past 50 years of the MRS professions. This includes, but is not limited to, the different generations of medical imaging equipment and hardware (computed radiography (CR) and digital radiography (DR) systems, magnetic resonance imaging (MRI) and computed tomography (CT) scanners, ultrasound probes, interventional radiology tools and linear accelerators), all of which aim to deliver safer, better, faster care for all patients.^3^, ^4^ Furthermore, new software updates hold promise for improved image acquisition, reconstruction and postprocessing, for improved image and therapy quality, higher diagnostic accuracy, precision medicine and better patient outcomes.^5^, ^6^, ^7^

There is an increasing recognition of the importance of patient needs and preferences expressed by recent movements like co‐production^8^, ^9^ and co‐design of health care. More effort and time have been subsequently invested in improving patient experience^10^ and comfort, preparing them for an examination and ensuring optimal communication before, during and after their visit in radiology or radiotherapy. Elaborate simulation programmes or suites, virtual reality and digital twinning interventions to prepare patients for scans or complex procedures are on the rise.^11^, ^12^ Given the increasingly culturally diverse populations encountered in clinical practice, different technological innovations are employed to improve accessibility and inclusion. Artificial intelligence‐enabled communicators, like the mobile‐based ones, described in the paper of Taylor and McLean^13^ are being trialled to ensure clear understanding of patient needs and preferences and to enable a safe and optimal examination, tailored to them. A variety of innovations explore ways to bring healthcare to the patient's bedside, minimise patient travelling and declutter the busy hospitals and clinical centres. These include wearable devices to monitor patient health, or the newly introduced mobile ultrasound or MRI equipment.^14^, ^15^

## The Rise of Artificial Intelligence: A New Friend or Foe on the Horizon?

The principle of artificial intelligence (AI) was established since the early 1950s and was initially practised in quiet computer laboratories.^16^ For many years, it remained the privilege and exclusive study reserved for the few, mainly computer scientists and biomedical engineers, who could understand and work with complex maths and stats. It took some years for AI to be brought from ‘the lab to the clinic’ and to start to be explored for clinical applications on patients.^17^, ^18^, ^19^, ^20^ There were three vital events that propagated this translation of AI to clinical practice: (1) the ‘big data’ era in the early 2000s, (2) the advancement of computational processing power, which led to the rapid digitisation of health care with, and (3) the increasing understanding of neuroscience on how the human brain learns and consolidates knowledge, setting the basis for machine learning in recent years.^21^ We are currently at an active stage of exploration, which will likely last for the next decade or so, until we have more robust data from AI implementation and real life use cases, to be able to determine the actual benefits and potential risks.^22^ Until this has happened, we cannot really be sure of the actual added value of AI for our patients, healthcare professionals and healthcare systems. We do already know, though, that AI is a form of disruptive innovation and requires robust testing both before application and after deployment.^23^, ^24^

## Embracing Technology‐Enabled Patient Care in MRS

All these new software and hardware technological solutions, whether AI‐enabled or not, are aiming to revolutionise the way health care in delivered, and the same holds true for the MRS profession. Medical radiation practitioners should be well prepared by nurture (education) and culture (professional identity) to adopt and work with latest technological developments. This could give them a unique advantage to assume roles as AI ambassadors, early adopters and leaders in diffusion of innovation in healthcare. It also creates a unique role of responsibility for every healthcare professional in MRS, in order to maximise benefits and minimise the risks of these new technologies. As these technologies are, often, untested, there needs to be centralised support for customised practitioner training and robust governance frameworks to offer guidance for technology deployment and adoption. In a well‐connected world, like the one we live in, a thoroughly evaluated technological solution will bring upon benefits for the whole of humanity and save thousands, potentially millions, of lives. This was the case of the latest mammography reporting software, that was recently disseminated in the United Kingdom (UK) and globally.^25^ Similarly, a poorly designed or inadequately tested technological innovation can create confusion and potentially harm patients and practitioners alike.^26^ Reliability, safety, reproducibility, patient consent, when using patient data, anonymity and confidentiality, all need to be thoroughly considered.^22^

## Harnessing Technological Innovation for Patient Benefit in MRS and Beyond

What is, therefore, required to be able to harness the benefits of technology to improve our ailing healthcare systems? How can healthcare professionals safely engage in that process, while abiding by the ‘do no harm’ principle?

1. *Co‐design and co‐production of innovation with key stakeholders*. Different studies show that there is huge value in co‐production and co‐design to ensure success for innovations in health care (and beyond).^8^, ^9^ These efforts should include both the practitioners and patients/public throughout the innovation lifecycle, from conceptualisation and design, testing, implementation and port‐market monitoring. The authentic contribution of end‐users can help refine the scope, ensure user‐friendliness and ergonomics, support safety, maximise accuracy, enhance transferability and interoperability, enable clinical efficacy and seamless deployment and adoption in clinical workflows and minimise the cost of any new technological intervention^7^, ^8^, ^9^ Including these key stakeholders early in their product development pipeline is not just the ‘ethical thing’ to do; the cost of the “afterthought” and exclusion of patients and practitioners is huge.^27^ Co‐production is not, and should not be, tokenistic; it is an essential stage of robust industrial design. So, medical radiation practitioners, members of the multi‐disciplinary team, patients and families/carers are essential resource in the development, testing and implementation of healthcare innovation, as experts by training, or experts by experience.
2. *Robust governance frameworks*. These could often be at national, or international level or involve some type of accreditation/evaluation standards relating to the safe use of new technologies. These can include standards for training, testing, deployment and post‐market monitoring and should be backed up by well‐designed research evidence. There are many national or international organisations that can do this, such as the British Standards Institute (BSI)^28^ or the International Organisation for Standardisation (ISO),^29^ and recently more work on this has been accentuated by the artificial intelligence sector and the different professional bodies and registration boards globally.^30^, ^31^
3. *Education and training* that is not just theoretical but also practical, for the new technologies that we are about to use and apply. This is vital, given that MRS professions are applied science professions, where consolidation of theory and translation of research materialise through clinical applications.^32^ The knowledge, skills and competencies necessary for diffusion of innovation by clinical practitioners will also need to be carefully considered in curriculum design and be evidence‐based.

## Conclusion

In the current healthcare landscape, there is an increase in technological advancements that can address our healthcare concerns and needs. However, it is imperative that we approach the development and testing of these innovations with the same level of caution, diligence and care that we have historically applied to new practices, tools or standards aimed at enhancing patient well‐being.^33^ Active engagement, robust governance and customised education are essential for us to contribute to this transformative era in health care.

## Conflict of Interest

The author declares no conflict of interest.

## Data Availability

Data sharing not applicable to this article as no new data were generated or analysed for this study.
